# Neuroaesthetic exploration on the cognitive processing behind repeating graphics

**DOI:** 10.3389/fnins.2022.1025862

**Published:** 2022-11-09

**Authors:** Yuan Qin, Lan Ma, Tuomo Kujala, Johanna Silvennoinen, Fengyu Cong

**Affiliations:** ^1^School of Biomedical Engineering, Faculty of Electronic Information and Electrical Engineering, Dalian University of Technology, Dalian, China; ^2^Faculty of Information Technology, University of Jyväskylä, Jyväskylä, Finland; ^3^School of Architecture and Fine Art, Department of Industrial Design, Dalian University of Technology, Dalian, China; ^4^School of Artificial Intelligence, Faculty of Electronic Information and Electrical Engineering, Dalian University of Technology, Dalian, China; ^5^Key Laboratory of Integrated Circuit and Biomedical Electronic System, Dalian University of Technology, Dalian, China

**Keywords:** event-related potentials, neuroaesthetics, perception, visual attention, graphic design

## Abstract

Repeating graphics are common research objects in modern design education. However, we do not exactly know the attentional processes underlying graphic artifacts consisting of repeating rhythms. In this experiment, the event-related potential, a neuroscientific measure, was used to study the neural correlates of repeating graphics within graded orderliness. We simulated the competitive identification process of people recognizing artifacts with graded repeating rhythms from a scattered natural environment with the oddball paradigm. In the earlier attentional processing related to the P2 component around the Fz electrode within the 150−250 ms range, a middle-grade repeating rhythm (Target 1) did not show a difference from a high-grade repeating rhythm (Target 2). However, in the later cognitive processes related to the P3b component around the Pz electrode within the 300−450 ms range, Target 1 had longer peak latency than Target 2, based on similar waveforms. Thus, we may suppose that the arrangement of the repeating graphics did not influence the earlier attentional processing but affected the later cognitive part, such as the categorization task in the oddball paradigm. Furthermore, as evidenced by the standard deviation wave across the trials, we suggest that the growing standard deviation value might represent the gradual loss of attentional focus to the task after the stimulus onset and that the zero-growth level may represent similar brain activity between trials.

## Introduction

Repeating graphics are ordered repetitions of similar graphics with aesthetic features. The use of repeating graphics is an essential modern design education method (Lupton and Phillips, 2008; Arntson, 2011). Repeating graphics, such as in Mondrian’s masterpieces and others, are generally used in the education of university students majoring in architecture and design (Kılıçaslan and Kuloglu, 2015). Moreover, repeating graphics are one of the essential methods for modern architecture and industrial product design, including the outstanding works from Zaha Hadid Architecture Studio (Sonderegger and Sauer, 2015; Whybrow, 2016; Bhooshan, 2017; El-Darwish, 2019). Repeating graphics are the basic patterns with cultural features used in graphic design (Cleveland, 2010; Quispel and Maes, 2014).

Until now, many studies about design education have focused on the design process and exploratory narrative processes (Ulusoy, 1999; Lee, 2009; Stones and Cassidy, 2010; Nicholas and Oak, 2020). Some researchers, such as Alexiou et al. (2009), have tried to combine the techniques from neuroscience and design to explore the neural correlates of participants’ design processes and feelings induced by designed products (Alexiou et al., 2009; Goucher-Lambert et al., 2019; Milovanovic et al., 2021). This study is a new attempt to explore the neural cognitive processes elicited by repeating graphics used in the design field. The measures of event-related potential (ERP) and event-related oscillation (ERO) from cognitive neuroscience were utilized for this exploration.

### Aesthetics and repeating graphics

Aesthetics as a discipline studies the aesthetic activities of people in the world of intentions and the variable levels of their effect on our daily emotions and experiences (Wassiliwizky and Menninghaus, 2021). At first, research on aesthetics was about fundamental psychology but it was not accepted as a mainstream branch of study until the appearance of neuroscience (Fechner, 1876; Zajonc, 1968; Berlyne, 1973). Neuroaesthetics, which adopts the research methods of neuroscience, was proposed by the French neurobiologist Semir Zeki and others (Kawabata and Zeki, 2004; Freedberg and Gallese, 2007; Chatterjee and Vartanian, 2016; Pearce et al., 2016).

**FIGURE 1:**
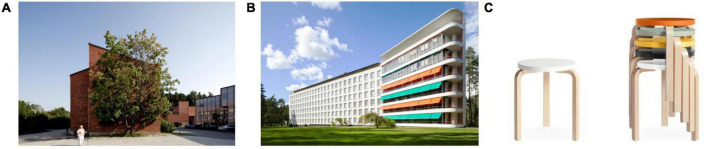
**(A)** University of Jyväskylä (Alvar Aalto, 1951–1971); **(B)** Paimio Sanatorium (Alvar Aalto, 1933a); **(C)** Model 60 stacking stool (Alvar Aalto, 1933b).

Repeating graphics are seen widely in art and design, with many aesthetic features. Appealing modern paintings such as Piet Mondrian’s Composition are constructed by repeating graphics (Kuspit et al., 1993; Deicher, 1999; Locher et al., 2005). Figure 1 shows the architecture and product design of Alvar Aalto (Finland, 1898–1976). The architecture of the University of Jyväskylä, as illustrated in Figure 1A (Alvar Aalto, 1951–1971), and the architecture of Paimio Sanatorium in Figure 1B (Alvar Aalto, 1933a) have repeating windows to maintain good illumination. The chairs in Figure 1C (Alvar Aalto, 1933b) can be piled quickly based on the use of repeating graphics in their design.

### Neuroscientific method

In neuroimaging methods, three parts of the electroencephalogram (EEG) method can be used in cognitive neuroscience. The first one uses spontaneous EEG recording, conducted without any accompanying external stimulus. The second is recording long-term natural stimuli, such as listening to music or watching sequential video images (Cong et al., 2013; Rogenmoser et al., 2016). The last is event-related potential (ERP), elicited by controlled stimuli (Handy, 2005; Luck, 2014). Compared to the two other EEG measures, the ERP signal-acquiring method enables researchers to study the cognitive process of brain-related features in specific categories. For the above reasons, ERP is the most suitable method for investigating brain processes induced by repeating graphics and other visual patterns. Moreover, the oddball paradigm is a typical way to record the ERP signal from the target stimulus (Polich, 2012). In the oddball paradigm, participants view stimuli in random sequences, consisting of about 80% standard stimuli and 20% target or deviation stimuli. In the experiment, participants press a button as quickly as possible when the target stimulus appears and do nothing for the appearance of standard or deviation stimuli. Every stimulus is presented on the screen for a short time, and a blank screen appears after the trigger in the interface (Demiralp et al., 2001; Moore et al., 2019; Teixeira et al., 2020; Sanada et al., 2021).

This study used simple stimuli with basic patterns and the ERP method in the oddball paradigm. It explored the primary neural correlates of repeating graphics in graded orderliness, such as their characteristics in drawing attention and the categorization procedure when the graphics were maintained in the working memory. Moreover, based on the superficial appearance of the stimuli, this study possibly correlated more with the earlier ERP components in cognitive processing, such as P2 and P300 (P3b). The P2 component is a positive ERP component and can be found approximately 200 ms after the appearance of a stimulus in the anterior and central parts of the brain (Luck and Hillyard, 1994). The P300 (P3b) component is the most studied endogenous component in the ERP family, and it has been found in a long and unstable time window of approximately 300 ms (Squires K. C. et al., 1975).

### Related objects for the brain research

In the oddball paradigm, there will be a significant P2 component, and it will also appear only when the stimulus is simple (Luck and Hillyard, 1994). One essential feature of the P2 component is that the P2 effect is enhanced when the target stimulus is infrequent and task-related. Regarding the perception features of the P2 component, it has been observed as an index that reflects the attention and discrimination process (Conley et al., 1999). It has been reported that the amplitude of P2 becomes larger if the stimulus is associated with more interest and attention (Eason, 1981; Mangun et al., 1986; Shedden and Nordgaard, 2001). For instance, Omoto et al. (2010) observed that the stimulus with a concave/convex feature motivated a larger P2 amplitude than a stimulus in a flat type. Stahl et al. (2008) observed that participants’ P2 response intensity was larger for own-race faces than seeing an other-race face. Participants with more experience communicating with other-race people did not show a difference in the amplitude of P2 between the two kinds of stimuli. However, the attention level for a stimulus should not be confused with the cognitive workload. Studies have found that the P2 amplitude decreased with the increased cognitive workload in a single-task paradigm (Allison and Polich, 2008; Deeny et al., 2014; Horat et al., 2016; Ghani et al., 2020). It meant that a complex stimulus might lead to decreased P2 amplitude compared to a simpler one. Another perception characteristic of the P2 component is its response to a repeated stimulus. Freunberger et al. (2007) designed a visual paradigm to observe the P2 signal feature based on two kinds of stimuli and found that P2 had a larger amplitude if the pair of stimuli were in different categories when they were shown in sequence. Other studies have observed the same kind of phenomenon in both visual and auditory fields (Wiggs and Martin, 1998; Rossell et al., 2003; Gruber and Muller, 2005). In addition, later research reported that facial images led to suppression in the P2 component when the face (stimulus) was of the same race as the participants’ (Sheng et al., 2016), and it supported the perception of repetition suppression. It was proposed that the repetition-related feature of P2 is an index that reflects long-term experience with prototypical features of the stimulus (i.e., stimulus features that appear more frequently in daily life).

Beyond the attention feature, P2 has been related to the emotional factors of a stimulus. The visual P2 component has been studied extensively in the area of lexicological psychology. Emotional words can modulate some kinds of ERP components in the earlier time window, such as P2 (Begleiter and Platz, 1969; Schapkin et al., 2000; Herbert et al., 2006). For instance, Kanske and Kotz (2007) found that the stimulus of a word associated with a positive emotion motivated a larger P2 amplitude than a neutral stimulus in a decision-making task. Moreover, similar studies have observed the same phenomenon (Kissler et al., 2006; Herbert et al., 2008; Schacht and Sommer, 2009).

The P300 (P3b) component is an endogenous component in ERP and is found in an unstable time window of approximately 300 ms. Squires N. K. et al. (1975) observed a component called the P3a component at the peak point around the frontal lobe and another one called P3b around the parietal lobe. Unpredictable and infrequent stimuli induce both of these components. However, P3b appears only when the stimulus is task-related, and P300 is usually used to refer to this P3b component. For instance, Kramer et al. (1995) observed that a task-irrelevant auditory stimulus did not generate the P300 (P3b) component. In addition, Miller et al. (2011) found that the P300 component would be more significant for novel stimuli, which grabs more attention than repetitive stimuli. This kind of feature of P300 was also reported by Dyke et al. (2015) in another study. Overall, P300 can be regarded as a measure of the cognitive distribution of attention.

Another exciting feature of P300 is that it has a smaller amplitude if a categorization task becomes more challenging. It has been reported that the amplitude of P300 decreases as the workload increases in an identity task (Goodin et al., 1983; Allison and Polich, 2008). Later, many studies have observed that P3 is related to higher-level cognitive processes such as categorizing stimuli and updating working memory. It has been reported that the latency of P300 represented the workload level and the categorization process. The stimulus was observed to have longer latency if it induced a higher workload. Participants also spent more time on the categorization task in this situation (Combs and Polich, 2006; Horat et al., 2016). The difference in the P300 (P3b) component latency between stimuli in the oddball paradigm meant completing the categorization task. The stimuli were categorized with different labels and stored in memory (Kutas et al., 1977). In addition, the latency of P300 increased with the participants’ age (Gaal et al., 2007).

Additionally, P300 is related to the emotional features of the stimulus. In earlier studies, many researchers have observed that the stimulus with more emotional features motivated a larger amplitude of P300 than a neutral one. Radilova (1982) reported that unpleasant visual stimuli produced a larger P300 amplitude than stimuli without an emotional response. Later, two other studies from Radilova et al. (1983), Radilova (1989) showed that sexual images motivated larger P300 amplitude than landscapes, flowers, and other stimuli which were not erotic. A survey from Muñoz and Martin-Loeches (2015) reported that the P300 increased when participants saw a beautiful stimulus compared to a neutral or negative stimulus. In addition, this phenomenon was primarily found around the frontal distribution.

As for the brain waves in the oddball paradigm and this research, the delta and theta waves are the most relevant. Delta waves are from 0 to 3 Hz (below 4 Hz), and theta waves are from 4 to 7 Hz. These two types of brain waves can be observed by EEG (Brigo, 2011), and it has been reported that delta and theta wave activity is related to the oddball paradigm. It has been reported that the theta waves respond more quickly than the delta waves in the P300 component. The theta waves were around the anterior lobe, while the delta waves were around the posterior lobe. Compared with the theta waves, the delta waves have been observed to be the most pronounced component correlated with the P300 wave (Demiralp et al., 1999). Moreover, the theta and delta waves are enhanced by presenting novel stimuli in the oddball paradigm, especially for the P300 amplitude. Many studies have observed that the anterior theta waves are related to preliminary cognitive processing and that posterior delta waves are relevant for later cognitive processing (Başar-Eroglu et al., 1992; Demiralp et al., 2001). For the type of stimulus, it has been observed that the theta and delta waves were enhanced for old (familiar) words compared to new (unfamiliar) words (Klimesch et al., 2000).

### Study overview

The current study simulates a procedure where people recognize graphic artifacts within different grades of repeating rhythm from scattered environments. This study was conducted with simple stimuli in graded orderliness in order to study the neural correlates of repeating graphics. The study was conducted using the event-related potential (ERP) measures and the event-related oscillation (ERO) in the oddball paradigm. The results of earlier components from ERP and ERO indicate significant differences between scattered graphics (as standard stimulus) and repeating graphics in different grades of orderliness (Target1 and Target2). The differences can be related to attention, short-term memory, long-term memory, or the categorization task.

## Materials and methods

### Participants

Twenty participants (9 female,11 male, mean age ± SD: 22.45 ± 2.41 years) were recruited by an intent questionnaire for a brain signal experiment at the Dalian University of Technology. The participants were right-handed based on the Edinburgh inventory (Oldfield, 1971) and had normal or corrected-to-normal vision. None of the participants had neurological disorders or used psychoactive medications. All participants were provided with informed consent in accordance with the Declaration of Helsinki (BMJ, 1991; 302:1194).

### Stimuli

**FIGURE 2:**
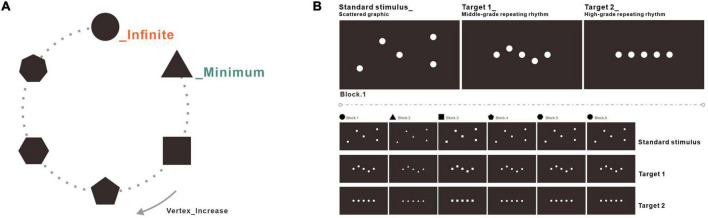
Stimuli used in the experiment. **(A)** The stimuli were composed of basic patterns with increasing vertexes, including circles, triangles, squares, pentagons, hexagons, or heptagons. **(B)** The standard stimulus was composed of scattered graphics with the lowest orderliness level. Target 1 was composed of the regular graphic within the middle-grade repeating rhythm. Target 2 was composed of a perfectly arranged graphic within the high-grade repeating rhythm.

The experiment consisted of three stimuli: standard stimulus, Target 1, and Target 2. The scattered graphic worked as the standard stimulus in the experiment. The graphic in a middle-grade repeating rhythm worked as Target 1, and the graphic in a high-grade repeating rhythm worked as Target 2 (see Figure 2). There were six blocks in the whole experiment, and the stimuli in every block were constructed by a specific basic pattern, including circles, triangles, squares, pentagons, hexagons, or heptagons. Taking block 1 as a reference, the standard stimulus was composed of scattered circles with the lowest repeating rhythm. Target 1 was composed of regular circles within the middle-grade repeating rhythm. Target 2 was composed of circles on a straight line, within the high-grade repeating orderliness. Every picture from the stimuli consisted of a black background and white graphics. The graphics in the experiment were in 1182 × 678 pixels with 300 dpi and 32-bit color. The details of the stimuli are shown in Figure 2.

### Procedure

**FIGURE 3:**
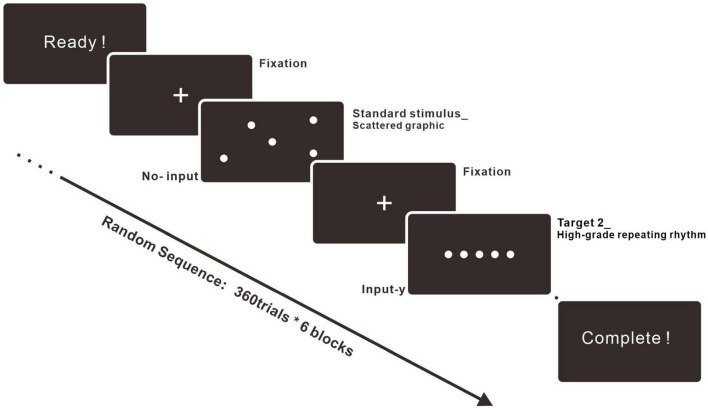
Experimental procedure. The experiment included six blocks. Each block consisted of 360 trials, and every trial was composed of fixation (600 ms), stimulus (1500 ms), and ISI (500 ms). The stimulus could be the standard stimulus (70%), Target 1 (15%), or Target 2 (15%). The trials were run in random sequence.

The ERP experiment was operated by an EEG recording device produced by the ANT Neuro company. The experiment followed the basic construction of the oddball paradigm, with a standard stimulus and two types of target stimuli (Demiralp et al., 2001). The experimental procedures were programmed and behavioral data such as response time (RT) was recorded by E-PRIME 3.0 (MacWhinney et al., 2001). Details about the procedures of the experiment are represented in Figure 3. The investigation was separated into six blocks. The standard stimulus, Target 1, and Target 2 blocks were composed of basic patterns with increasing vertexes, including circles, triangles, squares, pentagons, hexagons, and heptagons. Each block was run in two equal parts with a break in between to give participants a more relaxed experimental experience.

This study was designed to be an explorative but in-depth study of people’s neural responses to high-similarity stimuli. Thus, we designed an extended experiment with more trial numbers than other standard ERP research (Kappenman et al., 2021). For the experiment trials, there were 360 trials in each block, 2,160 trials for each participant, and 43,200 trials were collected in total. The standard stimulus accounted for 70% of every block for the experiment. Target 1 within the middle-grade repeating rhythm accounted for 15% and Target 2 within the high-grade repeating rhythm accounted for 15% of the stimuli. At the beginning of each block, there was an instruction to guide participants’ actions. Participants were recruited from the Dalian University of Technology in China, and therefore the words on the instruction were in Chinese, based on the participant’s native language. When the participants were ready, they pressed “SPACE” to start the block. Then, the trials related to the standard stimulus, Target 1 and Target 2, were displayed on the screen in a random sequence. Each trial consisted of three parts. The first part was the fixation, and it was presented for 600 ms. The next one was the stimulus. If the stimulus was the standard stimulus, participants were told to do nothing and wait for 1,500 ms. If the stimulus was Target 1 or Target 2, the screen was displayed again for 1,500 ms, but they were told to input “y” by the keyboard. The last part of the trial was the interstimulus interval (ISI), which lasted for 500 ms. After half a block or an entire block, there was a waiting page for a break, the length of which was based on participants’ preferences. After the break, they input “SPACE” to enter the next part.

### Data recording and processing

Electroencephalogram data were recorded with a 1,000 Hz sampling rate with a 64-lead EEG acquisition equipment produced by the ANT Neuro company and resampled to 256 Hz for further processing. The specific electrodes were Fp1, Fpz, Fp2, F7, F3, Fz, F4, F8, FC5, FC1, FC2, FC6, T7, C2, C3, Cz, C4, T8, CP5, CP1, CP2, CP6, P7, P3, P4, P8, Pz, POz, O1, O2, AF7, AF3, AF4, AF8, F5, F1, F2, F6, FC3, FCz, FC4, C5, C1, C2, C6, CP3, CP4, P5, P1, P2, P6, PO5, PO3, PO4, PO6, FT7, FT8, TP7, TP8, PO7, PO8, Oz, M1, M2, and CPz (online reference electrode). The EEG data were pre-processed by EEGLAB 2020 (Delorme and Makeig, 2004). The offline EEG data were re-referenced to the averages of the left mastoid (M1) and right mastoid (M2). Data from the additional EOG electrode were removed due to its lower correlation with this ERP study. Then, the line noise was removed by a notch filter of 49−51 Hz. In the next step, the wave band of data below 0.1 Hz was removed by a high pass filter, and the wave beyond 20 Hz was removed by a low pass filter after that (Lopez-Calderon and Luck, 2014; Widmann et al., 2015; Kappenman et al., 2021). Eye movement artifacts, electromyographic signal, and electro-cardio signal were rejected by the independent component analysis (ICA) (Jung et al., 2000a,b; Mognon et al., 2011).

The continuous EEG data were segmented into epochs (trials) based on the stimulus variety from −200 ms before the stimulus onset to 800 ms after the stimulus onset. The baseline correction was achieved by subtracting the mean amplitude of the baseline (from −200 to 0 ms) period from all time points. Bad trials were rejected by extreme value, and 78% of trials were reserved for each participant (about 1180 trials for the standard stimulus, 254 trials for Target 1 and Target 2). To obtain an equal trial number for the standard stimulus, Target 1, and Target 2, 254 trials from the standard stimulus were randomly selected from 1,180 trials by the “randperm” function in Matlab.

In the time domain analysis, we organized the data set based on trial numbers rather than participants due to a large number of trials per participant. By the average method in the ERP (in the time domain analysis), the final rendering wave did not show a difference in whether the data were averaged from trials directly to the grand average waveform, or first averaged from trials to participants and then to the grand average waveform. However, the ERP waveform organized into trials may express the statistical result more reliably due to the great number of trials. Thus, we collected the data into the fourth-order tensor. The index name of the tensor was channel*time*stimuli*trials, and the size was 61*256*3*5,080. In detail, each participant (20) had 254 trials for each stimulus. Afterward, the ERP waveforms of epochs were averaged based on the standard stimulus, Target 1, and Target 2, from 5,080 trials (Lopez-Calderon and Luck, 2014).

In the time-frequency domain analysis, we first organized the data by averaging the data into participants, as the conventional method. It should be noted that the time-frequency domain analysis could not be organized into trials because the power spectrum would be amplified more than ten times if the unstable single trial was processed by frequency transformation. Therefore, the statistical degrees of freedom (from the paired *t*-test) differed for the time domain analysis and the time-frequency domain analysis. The data became the fourth-order tensor, with the index name of channel*time*stimuli*participants, and the size was 61*256*3*20. In the following part, we computed the time-frequency representations (TFRs) of data averaged into participants based on the complex Morlet continuous wavelet transform (Tallon-Baudry and Bertrand, 1999; Roach and Mathalon, 2008; Cohen, 2014; Herrmann et al., 2014). Bandwidth and center frequency were set to define a complex Morlet used for the mother wavelet. The energies in the different frequency bands were obtained by calculating the square of convolutions between ERP signals, the shifted and scaled mother wavelet (Tallon-Baudry and Bertrand, 1999; Herrmann et al., 2005; Gross, 2014; Zhang et al., 2020b). The specific calculation was based on the toolbox of the ERP_ERO, and it can be downloaded from: http://zhangg.net/publications/ (Zhang et al., 2020a).

## Results

### Behavioral results

The participant’s task was to ignore the standard stimulus in the experiment and to respond to Target 1 and Target 2 with the keyboard. Regarding the behavioral data, the statistic of response time (RT) was calculated in the ERP experiment from six blocks (circles, triangles, squares, pentagons, hexagons, heptagons) and the data were categorized into Target 1 and Target 2. To count all of the experiments’ trials, the researchers constructed the RT data into a third-order tensor with the index name trial*block*stimuli.

**FIGURE 4:**
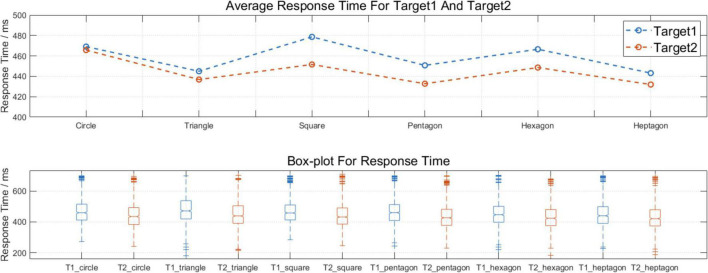
The behavioral results between blocks and within blocks. The mean response time (RT) for Target 2 was significantly shorter than the mean RT for Target 1, *t* (5079) = 13.75, ^***^p < 0.001, Cohen’s *d* = 0.37.

This research used a within-subject one-way repeated measure analysis of variance (rm-ANOVA) to analyze the significant differences between blocks and the paired *t*-test to explore the difference between RT data separated by Target 1 and Target 2. Details about the response times (RT) are shown in Figure 4. For the data between blocks, there were no statistically significant results. Importantly, when we organized the RT into Target 1 and Target 2, the mean response time (RT) for Target 2 was shorter than the mean RT for Target 1, and the significance of the difference was supported by the paired *t*-test, *t*(5,079) = 13.75, *p* < 0.001^***^, Cohen’s *d* = 0.37.

### The time domain analysis and the time-frequency domain analysis

**FIGURE 5:**
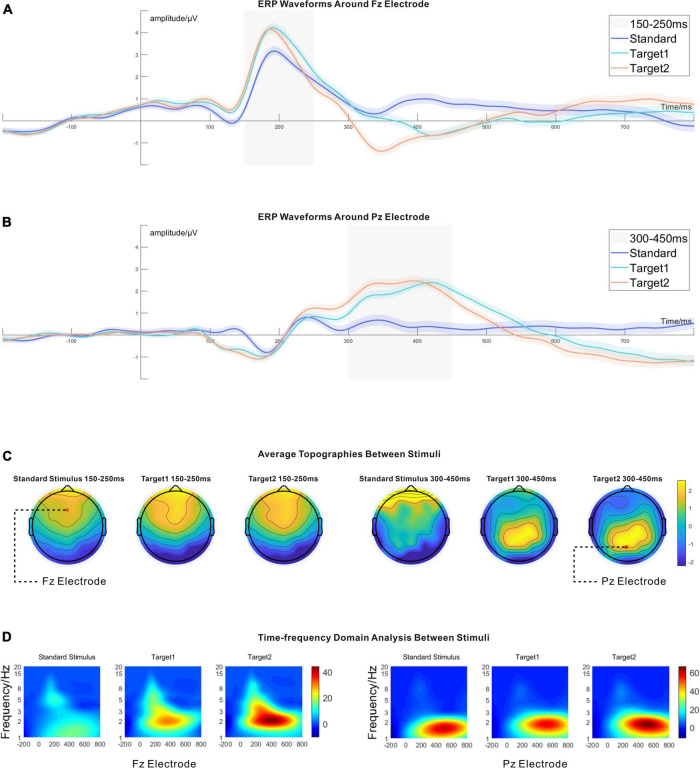
**(A)** The time domain analysis around the Fz electrode. There was no difference between Target 1 and Target 2 around the time window of 150–250 ms. The amplitudes of Target 1 and Target 2 were higher than the standard stimulus around 150–250 ms. The 95% CIs are shown as shadowed areas. **(B)** The time domain analysis around the Pz electrode. Likewise, no significant difference existed between Target 1 and Target 2 around the time window of 300–450 ms. Target 1 and Target 2 had higher amplitudes than the standard stimulus around the time window of 300–450 ms. The 95% CIs are shown as shadowed areas. **(C)** The topographies between stimuli around the time window of 150–250 ms and the time window of 300–450 ms. **(D)** The time-frequency domain analysis around the Fz electrode and the Pz electrode. Target 1 and Target 2 had higher power than the standard stimulus between 1 and 3 Hz, around the Fz electrode at 150–250 ms. However, there was no significant difference between Target 1 and Target 2 around the Fz electrode in 150–250 ms. Around the Pz electrode, only Target 1 and Target 2 had considerable differences at 300–450 ms. The statistical results are shown in Table 1.

In the 150−250 ms time window, a significant P2 component was found at the peak point around the Fz electrode (in the frontal lobe) for all stimuli. In the 300−450 ms time window, a significant P300 (P3b) component around the Pz electrode (in the parietal lobe) was found for Target 1 and Target 2. The results are shown in Figures 5A,B.

#### The time domain analysis and the standard deviation analysis

The fourth dimension was averaged from the fourth-order tensor (channel*time*stimuli*trials, 61*256*3*5,080), and the time domain analysis results resulted. Paired *t*-tests were run between stimuli for the time domain analysis (see Table 1). Around 150−250 ms, the Fz electrode showed significant P2 components for all stimuli in the time domain analysis. Target 1 and Target 2 had higher signal amplitude than the standard stimulus, but there was no significant difference between Target 1 and Target 2. The ERP wave around the Fz electrode is shown in Figure 5A. After establishing an average from 150 to 250 ms around the Fz electrode, the topographies are shown in Figure 5C, and the statistical results are shown in Table 1. Around the time window from 300 to 450 ms, there were significant P300 (P3b) components around the Pz electrode, induced by Target 1 and Target 2, and the ERP waveform around the Pz electrode is shown in Figure 5B. Likewise, the topographies averaged from 300 to 450 ms around the Pz electrode are shown in Figure 5C, and the statistical results are shown in Table 1.

**TABLE 1:** The paired *t*-test results for the time domain analysis and the time-frequency domain analysis.

Analysis type	Electrode time window	Matched pairs	Coupled difference					*t*	*Df*	Sig. (2 tails)	Cohen’s *d*
			Mean	*SD*	S.E. mean	95% CI					
						Lower bound	Higher bound				
Time domain analysis (5080 trials)	Fz electrode_average from 150 to 250 ms	Standard-target1	–0.90	8.06	0.11	–1.12	–0.68	–7.96	5079	< 0.001***	–0.22
		Standard-target2	–0.71	8.14	0.11	–0.93	–0.48	–6.17	5079	< 0.001***	–0.17
		Target1-target2	0.19	7.96	0.11	–0.02	0.42	1.77	5079	0.071	
	Pz electrode_average from 300 to 450 ms	Standard-target1	–1.49	9.52	0.13	–1.75	–1.22	–11.13	5079	< 0.001***	–0.31
		Standard-target2	–1.69	9.75	0.14	–1.96	–1.42	–12.35	5079	< 0.001***	–0.34
		Target1-target2	–0.20	8.35	0.12	–0.43	0.03	–1.73	5079	0.076	
Time-frequency domain analysis (20 subjects)	Fz electrode_average from 150 to 250 ms and 1 to 3 Hz	Standard-target1	–5.93	10.05	2.24	–10.64	–1.23	–2.64	19	0.016*	–1.21
		Standard-target2	–8.57	10.21	2.28	–13.35	–3.80	–3.76	19	0.001**	–1.73
		Target1-target2	–2.64	5.88	1.32	–5.39	0.11	–2.01	19	0.059	
	Pz electrode_average from 300 to 450 ms and 1 to 3 Hz	Standard-target1	–0.68	39.00	8.72	–19.38	18.02	–0.08	19	0.940	
		Standard-target2	–3.52	40.09	8.96	–22.28	15.24	–0.39	19	0.699	
		Target1-target2	–2.83	4.27	0.96	–4.83	–0.83	–2.96	19	0.008**	–1.36

The average values are from 150 to 250 ms around the Fz electrode and 300 to 450 ms around the Pz electrode. *p < 0.01 and 0.05, **p < 0.001 and 0.01, and ***p < 0.001.

**FIGURE 6:**
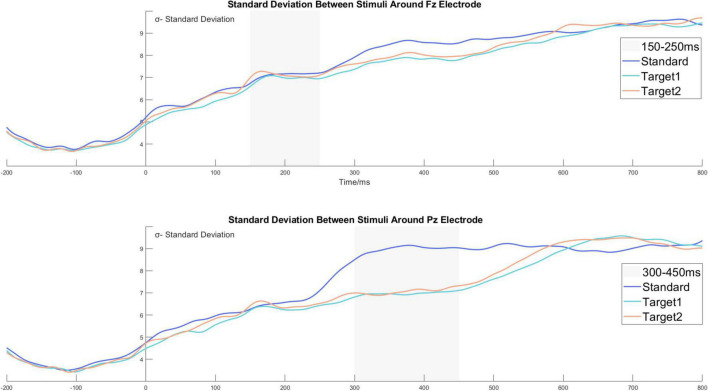
The standard deviation waves around the Fz and the Pz electrodes. The standard deviation value from a specific time point was calculated by the amplitude from 5080 trials. The standard deviation value represents the degree of dispersion under a particular channel, time point, and stimulus.

Furthermore, we relied on the data for the time domain analysis to calculate the dynamic standard deviation waveform between channels and stimuli. For illustration, under one channel and stimulus, the standard deviation value from a specific time point was calculated by the amplitude from 5,080 trials. The standard deviation value represented the degree of dispersion under a particular channel, time point, and stimulus. Details are shown in Figure 6. Moreover, we calculated the dynamic 95% confidence interval waves based on the existing standard deviation waves. Due to the excellent trial number (5080 trials for every stimulus), the dynamic 95% confidence intervals were tight, and the widest was about [μ − 0.28, μ + 0.28]. Figures 5A,B show the dynamic 95% confidence intervals as shadowed areas.

#### Time-frequency domain analysis

Corresponding to the time domain analysis, we also observed a significant difference in the time-frequency domain analysis around the Fz electrode in the time window of 150−250 ms and the Pz electrode in the time window of 300−450 ms between stimuli. Paired *t*-tests were again calculated between the stimuli (see Table 1). For the time-frequency domain analysis, Target 1 and Target 2 showed prominent event-related oscillation (ERO) without significant power difference in the low-frequency range from 1 to 3 Hz around the Fz electrode in 150−250 ms. However, the standard stimulus did not show a high-level frequency response. In the 300−450 ms time window, all stimuli showed high energy features from 1 to 3 Hz. Target 1 and Target 2 significantly differed in their frequency response around the Pz electrode, but there was no difference between the standard stimulus and Target 1 or between the standard stimulus and Target 2. The details are shown in Figure 5D, and the statistical results can be found in Table 1.

## Discussion

The current study investigated the brain signal response from stimuli within a repeating rhythm, which are essential elements of paintings, graphic design, and architectural design. The stimuli used in this study were basic and straightforward in appearance. They were represented as components in artwork with an aesthetic appeal (e.g., Deicher, 1999; Locher et al., 2005; Kılıçaslan and Kuloglu, 2015). The experiment simulated the process in which people recognize artifacts with repeating graphics from the natural environment. The scattered graphic was set as the experiment’s natural irregular graphic and a standard stimulus. Target 1 with a middle-grade repeating rhythm and Target 2 with a high-grade repeating rhythm needed to be recognized from scattered graphics with keyboard input. Moreover, it was a competitive recognition process between Target 1 and Target 2, because they all needed responses from participants. The study explored the neural correlates of the basic cognitive processes, focusing on attention grasping and other processes behind the perception of repeating graphics. The study intended to construct a foundation for future research that may set more complex art pieces composed of repeating graphics as the aesthetic objects in the experiment.

There were significant P2 components around the Fz electrode and the P300 (P3b) components were around the Pz electrode for the variable stimuli. These results were similar to earlier studies on P2 and P300 (P3b) with related topographies. Target 1 and Target 2 had a larger amplitude for the P2 component around the Fz electrode at the time window of 150−250 ms than the standard stimulus. Meanwhile, Target 1 and Target 2 showed considerable P300 (P3b) components compared to the standard stimuli at the time window of 300−450 ms around the Pz electrode (Luck and Hillyard, 1994; Kramer et al., 1995). The time-frequency domain analysis showed that Target 1 and Target 2 induced higher energy than the standard stimulus at a 150−250 ms time window, supporting the P2 components observed in the same time window. In addition, the wave feature of the time-frequency domain analysis in this study corresponded with earlier research, in that delta (0−3 Hz) and theta (4−7 Hz) waves were related to the deviating stimuli (Target 1 and Target 2 here) in the oddball paradigm. Meanwhile, the time-frequency domain analysis showed earlier theta waves around the anterior lobe and later delta waves around the posterior lobe for the three stimuli, also corresponding to earlier research (Başar-Eroglu et al., 1992; Demiralp et al., 2001).

### Earlier attention characteristics and later cognitive processes

Regarding earlier attentional processes around the time window of 150−250 ms, we observed that Target 1 and Target 2 showed a higher amplitude level of P2 than the standard stimulus, and that the P2 component amplitude between Target 1 and Target 2 was at the same level. Details are shown in Figure 5A and Table 1. These results are in line with earlier studies on the visual P2 component and its attention-related features (Wolach and Pratt, 2001; Lefebvre et al., 2005). As in the study of Luck and Hillyard (1994), the wave of the P2 component arose in the oddball paradigm for the infrequent stimuli. The P2 component seems only to appear when the stimulus is very simple (i.e., not complex in its appearance). The P2 component has been identified as an index to reflect an early discrimination process and the level of attention (Conley et al., 1999). Several studies have reported that the P2 amplitude becomes larger if the deviating stimulus grabs more attention from participants (Eason, 1981; Mangun et al., 1986; Shedden and Nordgaard, 2001). In our study, the higher P2 amplitude from Target 1 and Target 2 may be due to their low frequency of occurrence (15% both) in the oddball paradigm compared to the standard stimulus (70%).

Meanwhile, it was found that Target 1 and Target 2 showed no significant difference in the P2 component amplitude. The P2 component working as the earlier attention component is susceptible to the occurrence frequency in the oddball paradigm rather than the comparatively inconspicuous difference (same patterns in variable locations) between Target 1 and Target 2. In other words, the difference between Target 1 and Target 2 was insufficient to induce a considerable difference in the earlier visual attentional processing.

Moreover, several studies have reported that the amplitude of the P2 component decreases when the cognitive workload associated with a stimulus (i.e., its complexity) increases (Allison and Polich, 2008; Deeny et al., 2014; Horat et al., 2016; Ghani et al., 2020). Target 1 and Target 2 did not show considerable differences around the P2 component in this experiment. It further suggested that the difference in the arrangement between Target 1 and Target 2 did not have a statistical discrepancy in the earlier attention workload. Meanwhile, the complexity level and occurrence frequency are more devoted to the P2 component activation level, rather than merely the variable arrangement mode. Based on the above, we cautiously suggest that the aesthetic graphic pattern within the variable repeating arrangement may not significantly influence the viewer’s earlier attentional processes.

When it comes to later ERP components, the prominent P3b (P300) component from Target 1 and Target 2 gradually appeared. The ERP component we observed was consistent with earlier studies in that the P3b component was induced by a task-related target stimulus in need of a response, whereas the P3a component is induced by deviant without the need for responses from participants (Squires N. K. et al., 1975; Snyder and Hillyard, 1976; Jeon and Polich, 2001; Miller et al., 2011; Dyke et al., 2015). In our study, the scattered graphic worked as the standard stimulus, whereas the Target 1 and Target 2 stimuli were infrequently appearing and required participants’ responses. Both kinds of targets induced the P3b component around the Pz electrode by its task-related feature rather than the P3a component, which is irrelevant to the task and located around the Fz electrode.

For the time domain analysis results in the time window of 300−450 ms, we observed that the P3b components induced by Target 1 and Target 2 were significantly larger than the standard stimulus, and the average amplitude did not show a difference between Target 1 and Target 2. However, we observed that the P3b wave from the targets shared a similar wave appearance, but the waveform of Target 2 was a bit earlier than the waveform of Target 1, accompanied by different peak latency. To study the sustaining tracking phenomenon between Target 1 and Target 2, we calculated the dynamic 95% confidence interval for Target 1 and Target 2, shown as the shadowed areas in Figure 5B. The specific calculation method is described in the section “The time domain analysis and the standard deviation analysis,” and the standard deviation wave is shown in Figure 6. In Figure 5B, we can see the amplitude of Target 1 is smaller than that of Target 2 without the confidence interval overlap in the time window of 300−380 ms. The amplitude of Target 1 is then again larger than Target 2 in the time window of 420−500 ms. The potential correlation between the categorization task and the P3b may explain the phenomenon.

Earlier P300 (P3b) studies have reported that the peak point latency of P300 can represent the process of categorizing tasks of stimulus in long-term memory and attention allocation (Combs and Polich, 2006). Moreover, it has been widely accepted that the latency of P300 in the oddball paradigm is related to the completion of the categorization task and that the P300 (P3b) is the most famous endogenous component associated with the updating of working memory (Kutas et al., 1977; Combs and Polich, 2006; Horat et al., 2016). Based on the information mentioned, we supposed that participants spent more time on the classification task of the Target 1 stimulus with attention accompanied due to its longer P3b peak latency and persistently following the wave of Target 2. The behavioral data in Figure 4 show similar results: participants had a longer response time for Target 1 than for Target 2. Further, Target 1 showed lower energy power than Target 2 in the 1−3 Hz in the time-frequency domain analysis, which may be due to the same phenomenon.

For the experiment, we tried to simulate the cognitive processes of people recognizing artifacts from the scattered environment. Based on the results related to the P2 components around the Fz electrode and earlier research, we suggest that the different grades of arrangement in a repeating rhythm do not affect the earlier attention levels. However, the arrangement mode and the ambiguity level between the standard stimulus and the target stimulus in the oddball paradigm may influence the time required for the categorization task by later cognitive processes.

### The standard deviation wave and its tendency

As mentioned above, we calculated the dynamic standard deviation wave from the data based on trials, as shown in Figure 6. The original data set was organized as a fourth-order tensor, and the index name is channel*time*stimuli*trials (61*256*3*5080). By computing the 95% confidence interval, we obtained the dynamic confidence interval for the time domain analysis and depicted it as a shadowed area, as shown in Figures 5A,B. The confidence interval area may support the analysis results and assist in distinguishing the actual difference between waves in the time domain analysis. Compared to the related method of standard error measurement (Kappenman et al., 2021), the confidence interval area may have a high-level confidence coefficient and thereby improve the accuracy of the data.

We observed an interesting phenomenon from the standard deviation wave. Due to the baseline correction procedure from −200 to 0 ms, we only discuss the time zone after stimulus onset. In Figure 6, we observe that the standard deviation value continued to increase after the appearance of the stimulus. The standard deviation value started around 5 and increased to 9 at 800 ms. The growing standard deviation value (σ) may represent the gradual loss of attentional focus in a task after the stimulus onset. Furthermore, we observed that σ keeps a stable zero-growth level for all stimuli around the Fz electrode at 150−250 ms, and that the σ keeps the zero-growth level for Target 1 and Target 2 around the Pz electrode at 300−450 ms. However, the σ from the standard stimulus grew in advance and maintained a higher value than Target 1 and Target 2 around the Pz electrode at 300−450 ms. The zero-growth level time window was highly correlated with the P2 and P3b time window, and we suppose it also reflected a specific brain activity. Thus, with caution, we put forward a hypothesis that the ERP components may be due to a similar level of amplitude related to the settled time window, and that it contributes to the zero-growth level time in the standard deviation wave. We doubt whether the constantly growing standard deviation value and zero-growth level around the time window of the ERP component are a result of the large number of trials collected in our experiment. Other evidence is still needed to provide further support for the suggested phenomenon.

## Conclusion

To summarize, we tried to simulate the cognitive processes where people recognize graphic artifacts within different repeating grades from the scattered environment. In the earlier attentional processing related to the P2 component around the Fz electrode, a middle-grade repeating rhythm (Target 1) did not show a difference from a high-grade repeating rhythm (Target 2). By this evidence, we suggest that mere changes in the arrangement mode of the repeating rhythms do not affect the earlier attention features in oddball tasks. In the later cognitive processing related to the P3b component around the Pz electrode, Target 1 had a similar wave appearance to Target 2, but Target 1 had a longer peak latency than Target 2. It suggests that Target 1 needed a longer categorization time than Target 2, supported by the P3b component’s features and the longer response time for Target 1 in the behavioral results. Thus, we suppose that the arrangement mode of repeating rhythms in stimulus may not show a significant difference in earlier attentional processes but can affect later cognitive processing, such as the categorization task in the oddball paradigm. Furthermore, by observing the dynamic standard deviation waveform across trials, we suggest that the growing standard deviation value may represent the gradual loss of attentional focus after the stimulus onset and that the zero-growth level may represent similar brain activity across the trials.

Repeating graphics is one of the essential pattern types in modern art and design. Simple types of repeating graphics are elements of many famous art pieces like Piet Mondrian’s Composition. This study focused on stimuli with simple repeating graphics and their associated neural characteristics. We hope these findings may add helpful information for the research in the field of neuroaesthetics.

## Data availability statement

The raw data supporting the conclusions of this article will be made available by the authors, without undue reservation.

## Ethics statement

The studies involving human participants were reviewed and approved by the School of Biomedical Engineering, Faculty of Electronic Information and Electrical Engineering, Dalian University of Technology. The patients/participants provided their written informed consent to participate in this study.

## Author contributions

YQ, LM, and FC contributed to the conception and design of the study. YQ, TK, and JS organized the database. YQ and TK performed the analysis and wrote sections of the manuscript. YQ wrote the first draft of the manuscript. All authors contributed to the manuscript revision, read, and approved the submitted version.
